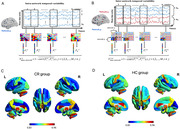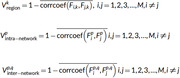# Dynamic Resting State Functional Connectivity in Cognitive Resilience of Alzheimer's Disease

**DOI:** 10.1002/alz.093493

**Published:** 2025-01-09

**Authors:** Lin Liu, Yalin Zhu, Anqi Li, Lili Fang, Shaohua Ma, Tengfei Guo

**Affiliations:** ^1^ Tsinghua Shenzhen International Graduate School (SIGS), Tsinghua University, Shenzhen China; ^2^ Institute of Biomedical Engineering, Shenzhen Bay Laboratory, Shenzhen, Guangdong China; ^3^ Institute of Biomedical Engineering, Shenzhen Bay Laboratory, Shenzhen China; ^4^ Institute of Biomedical Engineering, Peking University Shenzhen Graduate School, Shenzhen China

## Abstract

**Background:**

Alzheimer's disease (AD) is characterized by a decline in cognitive abilities, with cognitive resilience (CR) denoting the capacity of AD patients to withstand such declines. Prior studies have linked the segregation of functional networks with cognitive resilience in AD. The emergence of dynamic functional connectivity (dFC) is a notable advancement in the assessment of brain network dynamics of CR features in AD.

**Methods:**

Our present study adopted a novel metric (temporal variability), which has been successfully applied to many clinical diseases, to examine the dynamic functional connectivity of CR features in 164 AD participants into reference group of 96 with cognitive unimpairments (CU) and CR group of 68 with high CR. The specific calculation formula is in Figure 2, because the formula cannot be added to the text.

**Results:**

Figure 1 A‐B illustrates the flow chart used to calculate the separation coefficient within and between brain networks. Upon comparing the high cognitive elasticity group with the control group, we observed a significant decrease in the separation coefficient within and between the SMN, DMN, SUB, and CN brain networks in the high cognitive elasticity group (Figure 1C‐D).

**Conclusions:**

Through dFC features pertinent to CR can be obtained and have been corroborated in different clinical diagnosis groups and cognitive function scores.